# The association between Parkinson’s disease and temporomandibular disorder

**DOI:** 10.1371/journal.pone.0217763

**Published:** 2019-06-14

**Authors:** Ya-Yi Chen, Hueng-Chuen Fan, Min-Che Tung, Yu-Kang Chang

**Affiliations:** 1 Department of Stomatology, Tungs’ Taichung MetroHarbor Hospital, Wuchi, Taichung, Taiwan, Republic of China; 2 Institute of Genomics and Bioinformatics, College of Life Sciences, National of Chung Hsing University, Taichung, Taiwan, Republic of China; 3 Department of Pediatrics, Tungs’ Taichung MetroHarbor Hospital, Wuchi, Taichung, Taiwan, Republic of China; 4 Department of Medical Research, Tungs’ Taichung MetroHarbor Hospital, Wuchi, Taichung, Taiwan, Republic of China; 5 Department of Rehabilitation, Jen-Teh Junior College of Medicine and Management, Hou-Loung Town, Miaoli, Taiwan, Republic of China; 6 Department of Surgery, Tungs’ Taichung MetroHarbor Hospital, Wuchi, Taichung, Taiwan, Republic of China; 7 Department of Nursing, Jen-Teh Junior College of Medicine and Management, Hou-Loung Town, Miaoli, Taiwan, Republic of China; Chinese Academy of Sciences, CHINA

## Abstract

The prevalence of temporomandibular disorder (TMD) among elderly people with Parkinson’s disease (PD) is relatively high, but a population-based study of the relationship between PD and TMD is still lacking. This study, therefore, sought to investigate the association between TMD and PD by using data for one million randomly sampled beneficiaries of Taiwan’s National Health Insurance program, including 6,185 PD patients who were matched through propensity score matching with 18,555 non-PD patients. Both the PD and non-PD cohorts were followed until death, any diagnosis of TMD, or December 31, 2013, whichever occurred first. Each diagnosis of TMD was made by a qualified physician according to the International Classification of Diseases, 9th Revision, Clinical Modification (ICD-9-CM), using the diagnosis codes 524.60, 524.62, 524.63, and 524.69 while excluding tooth abscess, wisdom tooth eruption, herpes zoster and postherpetic neuralgia, mastoiditis, otitis externa, otitis media, parotitis, sialadenitis, and trigeminal neuralgia. We used Cox proportional hazard regression models to calculate the relative risk of TMD and found a 2.11-fold (95% CI: 1.35–3.30) increased risk of TMD overall in the PD group compared with the non-PD group. Stratified by follow-up period, there was a 4.25-fold (95% CI: 1.51–11.93) increased risk in the PD group in the first year after the initial PD diagnosis and a 3.88-fold (95% CI: 1.33–11.28) increased risk in the second year. Over the long-term (>5 years), PD was significantly associated with an increased risk of TMD. These findings suggest that it is important to closely monitor the temporomandibular joint health of PD patients.

## Introduction

Among the neurodegenerative disorders, Parkinson’s disease (PD) is second only to Alzheimer's disease in terms of prevalence, affecting 0.3% of the population in general [1] and more than 1% and 4% of people aged at least 60 and 80 years old, respectively [2–4]. PD is a chronic, progressive central nervous system disease that results from dopamine deficiency in the brain due to the death of dopaminergic neurons in the substantia nigra [5]. The resulting motor symptoms include postural instability, hand and facial tremors, muscular rigidity, impaired balance, and bradykinesia [6, 7]. Common postural instabilities seen among patients with PD include cervical spine flexion, thoracic hyperkinesis, scoliosis, abduction of the shoulders, and flexion of the arms [8].

The temporomandibular joint (TMJ) is a synovial joint containing an articular disk and allowing for hinge and sliding movements. Such complicated movements are necessary for chewing, swallowing, and speaking. The surfaces of the joint are covered by an avascular and noninnervated fibrous connective tissue. Historically, temporomandibular disorder (TMD) has been regarded as a localized condition of orofacial pain and dysfunction stemming from damage to the TMJ caused by abnormal occlusion, parafunctional habits (e.g., bruxism, teeth clenching, lip biting), stress, anxiety, or abnormalities of the intra-articular disk [9]. More recent scholarship, however, has come to view TMD as a complex biopsychosocial disorder influenced by a variety of genetic and environmental factors [10, 11]. Relatedly, TMD and a number of other pain conditions, such as fibromyalgia and chronic pelvic pain, are collectively described as chronic overlapping pain conditions (COPCs) due to the fact that they have been found to commonly occur together, both in individuals and in families [12, 13]. It is perhaps unsurprising, then, that a recent study of TMD patients found that such patients have higher prevalence rates of pain in other joints of the body than individuals without the disorder [14].

According to the results of the recent OPPERA prospective cohort study, TMD has an approximate annual incidence rate of 4% and is influenced by a wide range of risk factors in addition to clinical orofacial factors, including health status, psychological functioning, sociodemographic characteristics, health status, and pain sensitivity, among others [15]. Furthermore, while TMD and other COPCs predominantly affect women of childbearing age [10], various studies have found that TMD affects at least small percentages of the older populations most commonly affected by PD [16, 17]. Meanwhile, a recent study of PD patients found a significant relationship between PD and bruxism, as well as a likely relationship between PD and TMD pain [18].

TMD can present with a variety of clinical symptoms including joint noises, muscle pain, jaw locking, facial pain, neckaches, headaches, facial muscle fatigue, limited mouth opening, mandibular deviation, toothaches, dizziness, earaches, hearing problems, upper shoulder pain, and tinnitus [19]. A previous study also showed that there is a negative association between bite forces and the presence of TMD [20]. Meanwhile, the chronic, progressive nature of PD leads to impaired motor control, which has a negative impact on the maintenance of adequate oral hygiene and likely accounts for some of the impacts on oral health seen among individuals with PD [21–23].

A previous study of patients with cranial-cervical dystonia, including PD patients, found that such patients present a substantially higher risk for developing bruxism due to the progression of motor symptoms, while individuals with bruxism are more likely, in turn, to develop TMD [24]. A related study reported significant effects of TMD in PD patients in terms of functional limitations, psychological discomfort, and physical incapacity, including a negative impact on the physical activities of daily life, especially with regard to the functions of the stomatognathic system [25].

In spite of the apparent connection between PD and TMD indicated by previous studies [18, 24, 25], many of those studies had small sample sizes (n<100), cross-sectional designs, and a focus on evaluating the prevalence of the conditions; as such, a population-based study of the relationship between PD and TMD with a large sample size is still lacking. The aim of this study, therefore, was to use population-based data to compare the incidence of TMD in patients with PD to its incidence in matched controls. We also examined the relationship between the disorders among different subgroups based on different demographics, comorbidities, and observation periods. It was hypothesized that PD patients would exhibit a significantly higher incidence of TMD than matched non-PD patients.

## Materials and methods

### Data source

The National Health Insurance (NHI) program of Taiwan was started in 1995 and currently covers roughly 99% of Taiwan’s population of approximately 23 million people [26]. Beginning in 1999, the NHI program began to make its data available to researchers in the form of the National Health Insurance Research Database (NHIRD), which contains registration files and claims data for NHI beneficiaries. Specifically, the NHIRD contains data regarding patients’ diagnoses, demographics, medication types, prescription dates, dosages, and prescription durations [27]. In the present study, we examined data for 1 million NHI beneficiaries randomly sampled from the larger NHIRD. The datasets analyzed in the current study are available in the longitudinal health insurance database (LHID) 2000 repository. The National Health Research Institutes (NHRI) maintains oversight of the claims data and generates scrambled random identification numbers for insured patients in order to protect their privacy. Because the NHIRD contains deidentified secondary data that were analyzed anonymously, the need for informed consent in this study was waived. We ask that readers refer to the NHRI website (http://nhird.nhri.org.tw/en/Data_Subsets.html) for more detailed information on these datasets. This study was approved by the institutional review board of Tungs’ Taichung MetroHarbor Hospital in Taichung, Taiwan (106015). All the protocols used in the study were performed in accordance with relevant guidelines and regulations.

**Fig 1:**
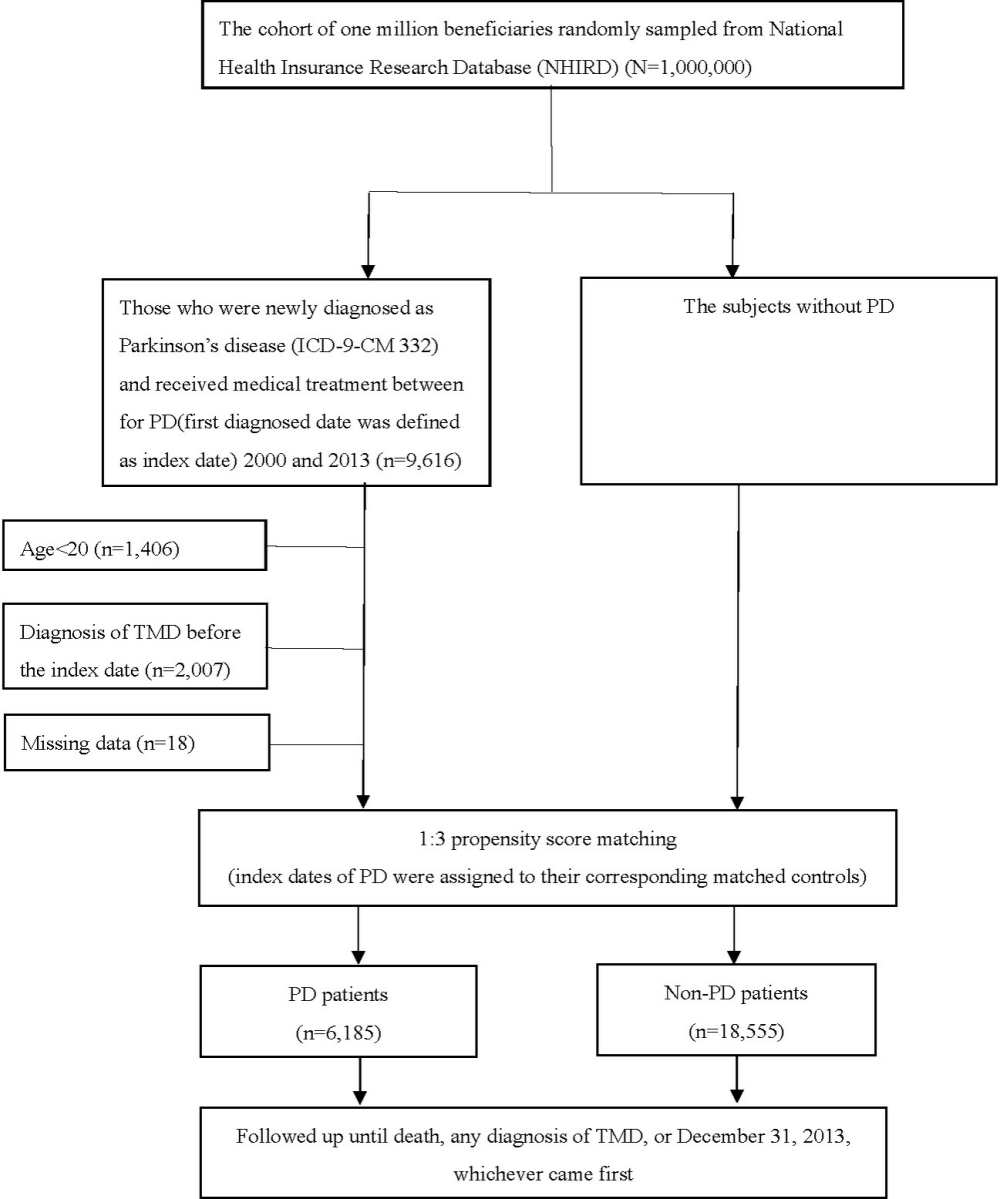
A flow chart of the patient selection process for this study.

From the one million NHI beneficiaries randomly sampled by the NHIRD, we first selected those who had received a diagnosis of PD from a qualified physician according to the International Classification of Diseases, 9th Revision, Clinical Modification (ICD-9-CM; code 332, n = 9,616), between 2000 and 2013. After excluding those aged less than 20 years old (n = 1,406); those already diagnosed with TMD (ICD-9-CM codes 524.60, 524.62, 524.63, or 524.69) following the exclusion of tooth abscess, wisdom tooth eruption, herpes zoster and postherpetic neuralgia, mastoiditis, otitis externa, otitis media, parotitis, sialadenitis, and trigeminal neuralgia before the index date (n = 2,007); and those who had missing data (n = 18), there were 6,185 PD patients remaining (Fig 1). These PD patients were then matched on a 1:3 basis with 18,555 non-PD patients, with the initial date of the PD diagnosis for a given PD subject being defined as the index date for the non-PD group subjects with which he or she was matched. In selecting the non-PD patients included in the comparison group, the 1:3 propensity score matching method [28] and the matched criteria based on the nearest neighbor algorithm with a perfect proportion of 0.995 to 1.0 [29] were used, with the propensity scores of all the study subjects being calculated by using multivariable logistic regression adjusting for age, gender, geographic region, and Charlson Comorbidity Index (CCI) [30]. The study subjects in both the PD and non-PD groups were followed until death, any diagnosis of TMD, or December 31, 2013, whichever came first.

### Definition of research variables

The main outcome of this study was the occurrence of TMD, which was defined as having received a diagnosis of TMD. TMD is a clinical diagnosis that must, in the NHI system, be made by an experienced physician based on a patient’s complaints, a physical examination, and the exclusion of tooth abscess, wisdom tooth eruption, herpes zoster and postherpetic neuralgia, mastoiditis, otitis externa, otitis media, parotitis, sialadenitis, and trigeminal neuralgia. The regular procedure in Taiwan for diagnosing TMD relies on the taking of a medical history, a physical examination, muscle palpation, a resistance test, arthroscopy, and a review of X-rays. The main comorbidities controlled for in this study included diabetes (ICD-9-CM code 250), hypertension (ICD-9-CM code 401), hyperlipidemia (ICD-9-CM code 272), and cardiovascular disease (ICD-9-CM codes 390–459). Study subjects were considered to have one of the above comorbidities if they had at least 2 ambulatory visits and 1 hospitalization associated with the respective diagnosis.

### Statistical analysis

The baseline data for the two groups are presented in terms of frequency, with percentages given for categorical variables and means with standard deviations given for continuous variables. T-tests and χ^2^ tests were used to describe the differences between the PD group and the non-PD group for the categorical variables and continuous variables, respectively. The incidence rate of TMD was defined as the number of events divided by the follow-up person-years, which were calculated as the time from the index date to the diagnosis of TMD, death, or December 31, 2013, whichever occurred first. We used Cox proportional hazards regression to determine the adjusted hazard ratios (aHRs) and 95% confidence intervals (CIs) of TMD for the PD group compared to the non-PD group. By conducting the Schoenfeld residuals test [31] and complementary log-log plots, we were able to confirm that the proportional hazards assumption was not violated. In the multivariable analyses, we adjusted for all the covariates shown in Table 1. To clarify the changes in TMD risk during the follow-up, we estimated the HRs of TMD in the following time periods: <1, 1–1.99, 2–2.99, 3–3.99, 4–4.99, 5–5.99, 6–6.99, and ≥ 7 years after the index date. All p values were 2-sided, and any *p*-value <0.05 was considered significant. All analyses were computed using SAS version 9.4 (SAS Institute Inc, Cary, North Carolina).

**Table 1:** The baseline characteristics of PD and non-PD patient groups.

	Case	Control	*p*-value χ^2^ test
Sex	n = 6185	n = 18,555	0.567
Female	3,021 (48.8)	8,989 (48.4)	
Male	3,164 (51.2)	9,566 (51.6)	
Age (in years)			
<60	1,091 (17.6)	3,405 (18.3)	0.258
60–70	1,233 (19.9)	3,833 (20.7)	
70–80	2,472 (40.0)	7,255 (39.1)	
80^+^	1,389 (22.5)	4,062 (21.9)	
mean±SD	70.2±13.5	70.0±13.7	0.304
Duration of follow-up	5.0±3.5	5.7±3.7	< .001
Geographic region			
Northern	2,466 (37.9)	7,410 (39.9)	0.053
Central	1,550 (25.1)	4,709 (25.4)	
Southern	1,986 (32.1)	6,006 (32.4)	
Eastern	183 (3.0)	430 (2.3)	
DM			
No	4,527 (73.2)	14,434 (77.8)	< .001
Yes	1,658 (26.8)	4,121 (22.1)	
Hypertension			
No	2,360 (38.2)	9,225 (49.7)	< .001
Yes	3,825 (61.8)	9,330 (50.3)	
Hyperlipidemia			
No	4,450 (71.9)	14,545 (78.4)	< .001
Yes	1,735 (28.1)	4,010 (21.6)	
Cardiovascular disease			
No	2,025 (32.7)	8,334 (44.9)	< .001
Yes	4,160 (67.3)	10,221 (55.1)	
CCI score			
0	1,796 (29.0)	5,489 (29.6)	0.609
1–5	2,932 (47.4)	8,790 (47.4)	
6^+^	1,457 (23.6)	4,276 (23.1)	
CCI score	3.65±3.00	3.58±2.95	0.102

Abbreviations: CCI: Charlson Comorbidity Index; DM: diabetes mellitus; SD: standard deviation

## Results

The PD patients, 51.2% of whom were male, had a mean age of 70.2 years and a mean CCI score of 3.65, with the largest percentage of these subjects (37.9%) living in the northern geographic region of Taiwan. The matched non-PD patients, 51.6% of whom were male, had a mean age of 70.0 years and a mean CCI score of 3.58, with the largest percentage of these subjects (39.9%) also living in the northern geographic region of Taiwan. The two groups were thus, as expected, similar in terms of the baseline characteristics on which they were matched (Table 1).

By the end of the 13-year period covered by the study, there had been a total of 32 incidences of TMD in the PD group (mean follow-up: 5.0 years) and 50 incidences of TMD in the matched control group (mean follow-up: 5.7 years). There was thus a significant difference in the incidence of TMD among the PD patients and the non-PD patients (p = 0.003) (data not shown). Overall, there was a 2.11-fold (95% CI: 1.35–3.30) increased risk of TMD in the PD group compared with the non-PD group after adjusting for age, sex, CCI score, geographic region, diabetes, hypertension, hyperlipidemia, and cardiovascular disease (Table 2).

**Table 2:** The aHRs of TMD in the PD patients compared with the non-PD patients.

	Crude HR (95%CI)	*p* value	Adjusted HR* (95%CI)	*p*-value for Cox proportional hazard model
PD				
No	Ref.		Ref.	
Yes	1.18 (1.40–3.39)	< .001	2.11(1.35–3.30)	0.001
Sex				
Female	Ref.		Ref.	
Male	0.57 (0.37–0.90)	0.005	0.61(0.39–0.95)	0.030
Age				
<60	Ref.		Ref.	
60–69	1.59 (0.86–2.93)	0.141	1.38(0.72–2.64)	0.325
70–79	1.00 (0.55–1.83)	0.996	0.88(0.45–1.69)	0.695
80^+^	0.52 (0.21–1.27)	0.149	0.46(0.18–1.16)	0.100
Geographic region				
Central & Eastern	Ref.		Ref.	
Southern	0.78 (0.47–1.27)	0.314	0.78(0.48–1.28)	0.330
Northern	0.54 (0.31–0.96)	0.034	0.56(0.31–1.00)	0.051
DM				
No	Ref.		Ref.	
Yes	1.10 (0.65–1.86)	0.712	1.03(0.59–1.80)	0.914
Hypertension				
No	Ref.		Ref.	
Yes	1.06 (0.69–1.64)	0.794	1.18(0.61–2.27)	0.625
Hyperlipidemia				
No	Ref.		Ref.	
Yes	1.36 (0.82–2.25)	0.237	0.94(0.53–1.65)	0.825
Cardiovascular disease				
No	Ref.		Ref.	
Yes	1.24 (0.80–1.93)	0.338	0.53(0.24–1.20)	0.128
CCI score				
0	Ref.		Ref.	
1–5	1.05 (0.63–1.81)	0.810	1.08 (0.62–1.86)	0.570
6^+^	1.17 (0.64–2.13)	0.601	1.24 (0.66–2.32)	0.112

*Adjusted for gender, age, geographic region, and CCI score.

Abbreviations: CCI: Charlson Comorbidity Index; DM: diabetes mellitus; HR: hazard ratio; Ref: reference group

**Fig 2:**
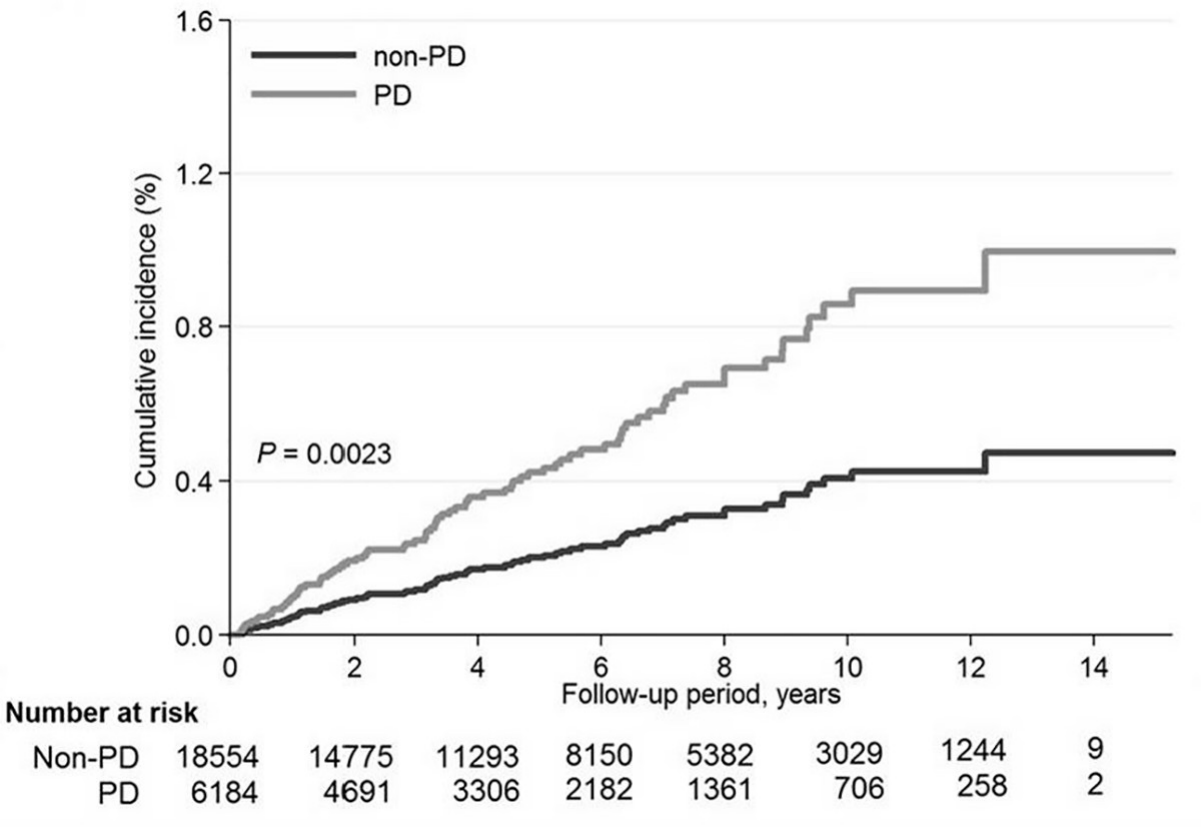
Cox proportional hazards regression analysis results for the cumulative incidence of TMD in the patients with PD and the matched controls, with adjustments made for age, sex, geographic region, Charlson comorbidity index, history of diabetes, hypertension, hyperlipidemia, and cardiovascular disease.

**Fig 3:**
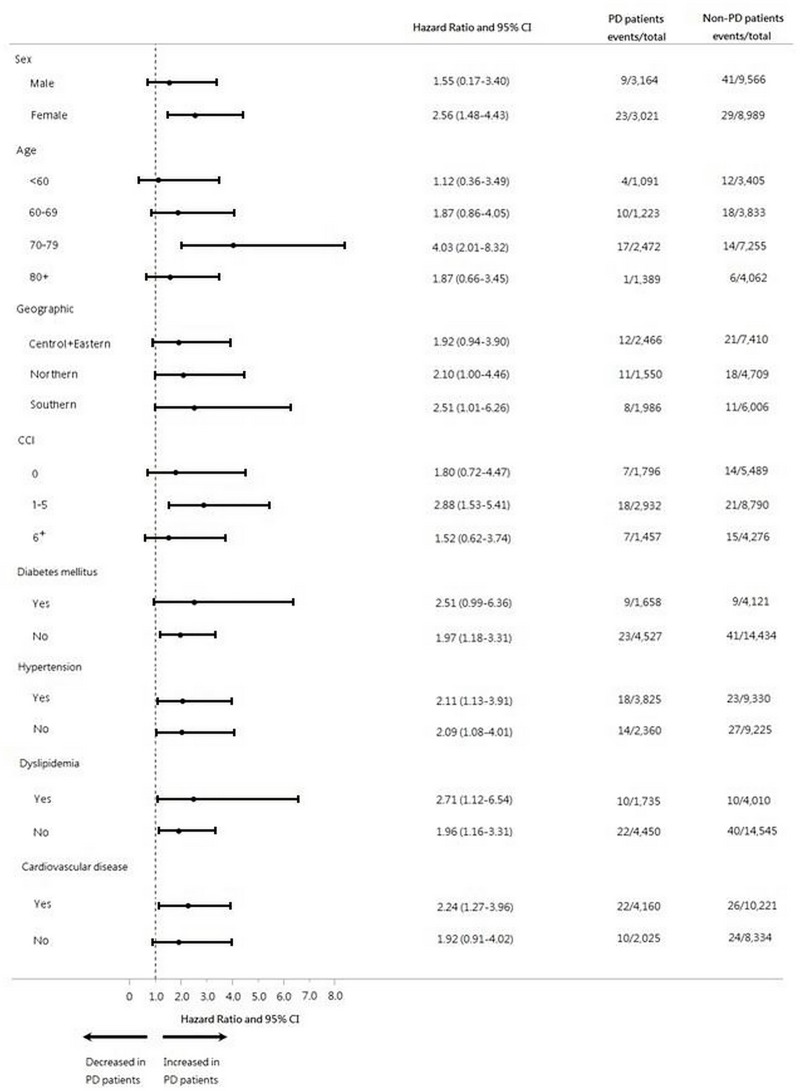
Risk of TMD in subgroups of matched patients.

According to the Cox proportional hazards regression analysis (Table 3; Fig 2), the incidences of TMD were 26.11 per 1000 person-years during the first year, 6.65 per 1000 person-years during the second year, 1.65 per 1000 person-years during the third year, 1.67 per 1000 person-years during the fourth year, and 9.26 per 1000 person-years after the first 4 years since the initial PD diagnosis for the PD group, compared with values of 6.43, 1.94, 0.90, 3.26, and 1.31, respectively, for the matched non-PD group. These results indicated significantly increased risks of TMD in the PD group in comparison to the non-PD group during the first year (aHR: 4.25, 95% CI: 1.51–11.93) and second year (aHR: 3.88, 95% CI: 1.33–11.28). The results of the subgroup analysis were thus consistent with the main finding. That is, in comparison with the non-PD group, the PD group exhibited an increased risk of TMD across all clinically relevant subgroups (Fig 3).

**Table 3:** Risk of TMD among the PD patients and the matched non-PD patients by different follow-up period.

The period since index date (years)	Events	Person-years of follow-up	Incidence rate (per 1,000 person-years)	Crude HR (95%CI)	*p*-value	Adjusted HR* (95%CI)	*p*-value for Cox proportional hazard model
<1							
Non-PD	6	933	6.43	Ref.		Ref.	
PD	10	383	26.11	4.08 (1.48–11.22)	0.007	4.25 (1.51–11.93)	0.006
1–1.9							
Non-PD	6	3,088	1.94	Ref.		Ref.	
PD	8	1,203	6.65	3.59 (1.24–10.35)	0.018	3.88 (1.33–11.28)	0.013
2–2.9							
Non-PD	4	4,437	0.90	Ref.		Ref.	
PD	3	1,820	1.65	1.90 (0.42–8.47)	0.402	1.95 (0.42–9.07)	0.397
3–3.9							
Non-PD	10	6,005	1.67	Ref.		Ref.	
PD	3	2,296	1.31	0.80 (0.22–2.90)	0.730	0.87 (0.24–3.17)	0.827
4–4.9							
Non-PD	5	7,274	0.69	Ref.		Ref.	
PD	1	2,789	0.36	0.54 (0.06–4.64)	0.576	0.54 (0.06–4.70)	0.577
5–5.9							
Non-PD	3	8,367	0.36	Ref.		Ref.	
PD	2	2,757	0.73	1.99 (0.33–11.93)	0.450	1.83 (0.29–11.45)	0.519
6–6.9							
Non-PD	5	9,408	0.53	Ref.		Ref.	
PD	2	2,981	0.67	1.26 (0.24–6.51)	0.781	1.18 (0.22–6.24)	0.845
≧7							
Non-PD	11	66,669	0.16	Ref.		Ref.	
PD	3	16,827	0.18	1.12 (0.31–4.00)	0.866	1.09 (0.30–3.95)	0.892

*Adjusted for gender, age, geographic region, and CCI score.

Abbreviations: HR: hazard ratio; Ref: reference group

## Discussion

In this study, we found a significantly increased risk of TMD among patients with PD as compared to non-PD patients, particularly among those patients who had lived with PD for an extended period of time. While sometimes undervalued or compartmentalized as separate from the health of the rest of the body, oral health is of critical importance given its numerous physical and psychological effects, including its impacts on how people look, speak, and socialize, among others [32]. By causing pain suffering, and alterations in diet, oral health problems can directly affect overall health, including people’s quality of life and well-being in general [33]. Moreover, oral health or the lack thereof can have direct effects on chronic diseases such as PD [34]. For example, various studies have reported associations between poor oral health and chronic obstructive pulmonary diseases, cardiovascular disease mortality, and respiratory disease mortality [35, 36].

At the same time, because certain chronic diseases can affect an individual’s ability to maintain proper oral hygiene, it is hardly surprising that the association between oral health and chronic diseases can go in the other direction–that is, that certain chronic diseases can lead to oral health problems. For example, a recent study of elderly subjects with Alzheimer’s disease found that they had worse oral health than elderly individuals without Alzheimer’s disease [37]. Relatedly, the findings of various studies indicating that PD, with its similarly profound impacts on activities of daily living, is associated with dental diseases and decay are arguably to be expected to some degree [22, 38]. Nakayama et al. reported, for example, that, in comparison to non-PD controls, PD patients were less likely to still have their own teeth or to clean their dentures every day [38]. Furthermore, because PD primarily affects the motor system, it is likely that the resulting motor symptoms have direct effects on the prevalence of TMD (that is, in addition to the indirect effects they have on oral health via their impacts on self-care activities).

In spite of the apparent connection between PD and TMD indicated by previous studies [18, 24, 25], however, the results of the present study provide the first population-based evidence of an increased risk for TMD among PD patients. Specifically, the PD patients were found to have a significantly increased incidence of TMD in comparison to the non-PD patients (p = 0.003) (data not shown), with an overall risk of TMD 2.11 times (95% CI: 1.35–3.30) higher for the PD group than for the non-PD group even after adjusting for age, sex, CCI score, geographic region, diabetes, hypertension, hyperlipidemia, and cardiovascular disease (Table 2). More specifically, the results indicated significantly increased risks of TMD in the PD group compared to the non-PD group during both the first year (aHR: 4.25, 95% CI: 1.51–11.93) and second year (aHR: 3.88, 95% CI: 1.33–11.28) after the index date of PD diagnosis.

The effects of TMD can be profound. For example, a systematic review of past studies regarding the effect of TMD on oral health-related quality of life (OHRQoL) found the effects of TMD on OHRQoL to be substantial, with its effect on pain being particularly notable [19]. With respect to psychological impacts, a study of Singaporean TMD patients conducted by Yap et al. found that 39.8% experienced moderate to severe depression, a substantially higher rate than the roughly 3% reported for the global population in general [39, 40]. In light of these direct and significant effects of TMD, and given that PD is itself associated with increased rates of depression [41], among other comorbidities, the results of the current study underscore the critical importance of checking for TMD in patients with PD.

There were some potential limitations to the present study. First, because a retrospective cohort study of this type is affected by various biases relating to the required adjustments for confounding factors, the data obtained from it does not have methodological quality as high as that of the data garnered from a well-designed randomized trial. Second, the instruments used to diagnose TMD were not standardized across all of the reporting sites. The diagnosis of TMD is clinical. However, only physicians who are familiar with the condition will make the diagnosis after excluding possible differential diagnoses, such as tooth abscess, wisdom tooth eruption, herpes zoster and postherpetic neuralgia, mastoiditis, otitis externa, otitis media, parotitis, sialadenitis, and trigeminal neuralgia. Therefore, there was no standardized diagnostic instrument for TMD used in this study. Third, and relatedly, the clinical examinations conducted for the diagnoses of TMD were not calibrated across all the examiners. Because this was a retrospective study, we could not ask all the physicians in the study to carry out the same clinical examination and make the diagnosis according to the exact same criteria. However, we only enrolled those patients who had been diagnosed with TMD at least three times according to the NHI data. Fourth, psychological illnesses that might potentially contribute to the inter-connection between PD and TMD, such as pica, were not considered in this study. Furthermore, although we performed propensity score matching and excluded pre-TMD history in both groups to minimize the impact of the measured covariates, our findings cannot demonstrate unobserved confounding factors including detailed information on potential confounding factors such as patient lifestyle, habits, socioeconomic status, physical activity level, family history, etc. As such, biases resulting from potential unknown and/or unmeasured confounding factors could have affected the results. Future studies could provide further confirmation and clarity regarding the long-term associations of PD with TMD by addressing some of the above limitations. For example, a future study using a standardized diagnostic instrument for TMD could provide more reliable data, while a future study considering the effects of psychological disorders that could potentially contribute to the relationship between PD and TMD would broaden our understanding of that relationship.

That said, the strengths of the study include its population-based design, with the use of population-based NHIRD records providing a large sample size of both PD and propensity-matched non-PD patients suggesting that the generalizability of the findings should be high. Relatedly, because the reimbursement policy of the NHI is universal and operated by a single buyer, that is, the government of Taiwan, the NHIRD provides a highly representative sample of the general population of Taiwan. Moreover, while the claims data contained in the NHIRD claims are primarily used for administrative, rather than scientific, purposes, which may affect their validity, the claims are generally peer-reviewed and checked by medical reimbursement specialists. As such, the data in the NHIRD regarding diagnoses have been found to be highly reliable in general. Otherwise, while retrospective cohort studies can be ineffective in providing evidence regarding rare disorders, and while the 0.3% prevalence of PD makes it more uncommon than many non-neurodegenerative diseases, the large population-based sample of the current study and the relatively higher incidence of PD among elderly individuals should have mitigated this limitation.

## Conclusions

In conclusion, the results of this study demonstrated that the PD patients had an increased risk of TMD compared to the matched controls, with the difference being significant for 2 years after the diagnosis of PD. Understanding these long-term associations of PD with TMD, as well as the risk of causal motor symptoms, is of substantial value for those seeking to improve oral health policies.
